# Genetic Evidence of Expansion by Passive Transport of *Aedes* (*Stegomyia*) *aegypti* in Eastern Argentina

**DOI:** 10.1371/journal.pntd.0004839

**Published:** 2016-09-01

**Authors:** Leonardo M. Díaz-Nieto, Marina B. Chiappero, Clara Díaz de Astarloa, Arnaldo Maciá, Cristina N. Gardenal, Corina M. Berón

**Affiliations:** 1 Instituto de Investigaciones en Biodiversidad y Biotecnología (INBIOTEC), CONICET, Mar del Plata, Argentina; 2 Fundación para Investigaciones Biológicas Aplicadas (FIBA), Mar del Plata, Argentina; 3 Instituto de Diversidad y Ecología Animal (IDEA), CONICET and Universidad Nacional de Córdoba, Córdoba, Argentina; 4 División Entomología, Facultad de Ciencias Naturales y Museo Universidad Nacional de La Plata, La Plata, Argentina; University of California, Davis, UNITED STATES

## Background

*Aedes* (*Stegomyia*) *aegypti* (Linnaeus) (Diptera: Culicidae) is the principal vector of the yellow fever virus, the five dengue virus serotypes (DENV-1 to DENV-5), chikungunya virus, Zika virus, and several types of encephalitis [1–3]. The behavior of this species is synanthropic and anthropophilic, being the culicid most closely associated with human populations [4]. The incidence of dengue has increased 30-fold over the last 50 years; according to the World Health Organization, up to 50–100 million infections occur each year in over 100 endemic countries, and at least one half of the world’s population has risk of being infected with dengue virus [5]. Chikungunya virus has been responsible for over 2 million human infections during the past decade and is currently moving to subtropical latitudes as well as to the western hemisphere. Up until April 2015, there have been 1,379,788 suspected cases of this disease in the Caribbean islands, Latin America, and the United States. This expansion into novel habitats brings unique risks associated with further spread of the virus and the disease it causes [6]. On the other hand, there are about 200,000 cases of yellow fever each year worldwide responsible for about 30,000 deaths, most of them from Africa. Zika virus is an emerging mosquito-borne virus, with outbreaks in Africa, Asia, and the Pacific between 2007 and 2014. Since 2015, there has been an increase in reports of ZIKV infection in the Americas, with Brazil being the most affected country, with 534 confirmed cases and 72,062 suspected cases between 2015 and 2016 [7]. All these viruses and the mosquito vector *A*. *aegypti* present in the Americas represent a serious risk. So far, in 2016, 39,926 dengue cases produced by DENV-1 and DENV-4 serotypes and 319 autochthonous cases of chikungunya fever have been reported in Argentina. According to the last census, Argentina has approximately 40 million people (National Institute of Statistics and Censuses of Argentina [INDEC], Census 2010), and over 38 million live in areas suitable for the transmission of dengue and chikungunya viruses [8]. Moreover, although there were 22 imported cases of Zika and 24 autochthonous cases confirmed in Argentina, there is a high incidence of cases in Brazil, and besides that, there is an internal circulation of the virus in the neighboring countries Brazil, Paraguay, and Bolivia [7,8], in addition to the constant expansion of the mosquito vector [9].

In particular, Argentina participated in the *A*. *aegypti* eradication program carried out by the Pan-American Health Organization between the 1950s and early 1970s; the species was eradicated from the country in 1967. However, in 1986 the mosquito was detected in the northeast of the country in the border with Paraguay, and some years later, it was found in northwestern and central areas. As a consequence, the current geographical distribution of the species is wider than before its eradication [10,11], with a fast and constant expansion to the south [9].

Albrieu Llinás and Gardenal [12] demonstrated that a 450-bp fragment of the mitochondrial nicotinamide adenine dinucleotide hydride (NADH) dehydrogenase subunit 5 gene (ND5) was a reliable marker to estimate the genetic structure of *A*. *aegypti* populations in Argentina. In this phylogeographic study, they detected 14 haplotypes from 22 populations covering most of the distribution of this species in the country, identifying three main haplogroups that suggest different colonization events from neighboring countries: Bolivia, Paraguay, and Brazil. The authors proposed that the absence of genetic variability in the east of Argentina and Paraguay was due to successful mosquito eradication campaigns, with recent recolonization of the region by founder events followed by a rapid range expansion. On the other hand, inefficient control campaigns in the northwest of Argentina would have caused the maintenance of relictual populations, resulting in high haplotype variability in the area [13].

**Fig 1:**
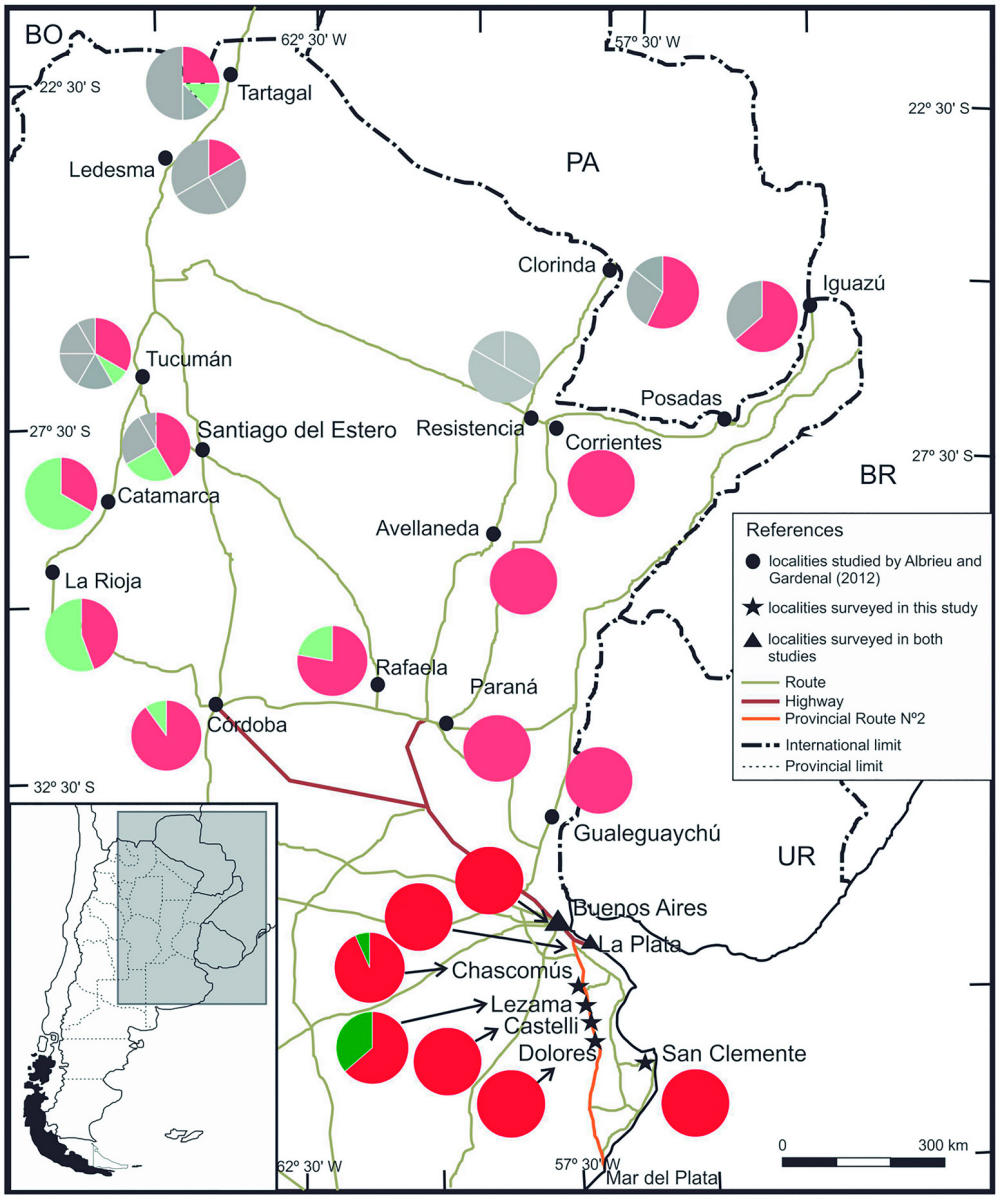
Haplotype frequencies for ND5 gene in studied populations and comparison with some of the populations analyzed by Albrieu Llinás and Gardenal [12]. Frequencies for H1 are indicated in red, and for H2, in green. Populations analyzed by Albrieu Llinás and Gardenal [12] are included for comparison purposes; only frequencies for H1 (light red) and H2 (light green) are indicated; all other haplotypes are in grey. Haplotype frequencies in the cities of Buenos Aires and La Plata were the same as in Albrieu Llinás and Gardenal [12]. Bordering countries: BO, Bolivia; PA, Paraguay; BR, Brazil; UR, Uruguay.

In 2002, the southernmost limit for *A*. *aegypti* in Argentina was Chascomús, 130 km away from the city of Buenos Aires (Fig 1) [14]. Then, in 2013 our group studied the biogeographical record of *A*. *aegypti* in the southeast of the country, confirming the last record of this species in Chascomús but also finding the species between March 2011 and 2012 in towns along Route Number (N°) 2 for the first time, specifically at the city of Dolores, which is 98.7 km from Chascomús (Fig 1) [15]. Recently, Zanotti et al. [9] reported Villa Gesell, a small town on Provincial Route N° 11 at 110 km from Mar del Plata and 376 km from the city of Buenos Aires, as the southernmost limit.

Most of the areas sampled by our group in 2013 are on the side of one of the most important highways in the country, Route N° 2, which connects the cities of Buenos Aires and La Plata with the city of Mar del Plata and the most visited coastal towns. This highway takes the bulk of the traffic in a southeastern direction, concentrating the majority of people who move from the north of Argentina and the neighboring countries to the coast, principally in the summer time [16]. Despite the fact that this mosquito is well established in small towns along Route N° 2, such as Dolores as well as to the north of Argentina and in the neighboring countries, the presence of *A*. *aegypti* was not reported for Mar del Plata.

The aim of this report is to determine the origin of the *A*. *aegypti* populations along Route N° 2, analyze the mitochondrial lineages, and compare their haplotypes with the haplotypes previously determined by Albrieu Llinás and Gardenal [12] in Argentina and neighboring countries.

## Haplotypes Present in the Southern Biogeographic Distribution of *A*. *aegypti* in Argentina

All through March 2013, mosquito larvae were collected and identified as *A*. *aegypti* according to a specific key [14]. The sampling stations were cemeteries (flower pots) and used tires located in the towns next to the Provincial Route N° 2 (Lezama, Castelli, and Dolores); additionally, we took into consideration the cities of Buenos Aires and La Plata (both at approximately 400 km north from Mar del Plata) and San Clemente del Tuyú, a small town located on the Atlantic coast on the Provincial Route N° 11 (at 328 km south from the city of Buenos Aires). New samples from Buenos Aires and La Plata were analyzed in order to confirm if the haplotypes detected by Albrieu Llinás and Gardenal in 2012 are still present in those places and, therefore, if the new results could be compared. The sampling station from San Clemente del Tuyú was the only one obtained by our group in Route N° 11 until 2013 (Fig 1). To reduce the risk of including individuals from eggs coming from the same female, the insects were collected from three or four different larval habitats in each sampling site analyzed, except for San Clemente del Tuyú, where only one place was positive for *A*. *aegypti* larvae (Table 1).

**Table 1:** Sampling stations and specimens of *A*. *aegypti* collected.

Town	Number of Flowerpots Revised	Number of Used Tires Revised	Number of Individuals Obtained per Town	Number of Individuals Analyzed per Population
Chascomús	200 (0)^a^	28 (12)^a^	315	15
Lezama	200 (0)^a^	31 (15)^a^	264	12
Castelli	300 (0)^a^	18 (9)^a^	230	16
Dolores	450 (0)^a^	23 (13)^a^	367	11
Avellaneda	n/d^b^	15 (10)^a^	210	11
La Plata	n/d^b^	20 (13)^a^	346	11
S. Clemente^c^	n/d^b^	5 (1)^a^	125	10

^a^ In parentheses, positive sampling stations for *A*. *aegypti*.

^b^ n/d, no data.

^c^ S. Clemente, San Clemente del Tuyú.

Total DNA from a single mosquito at the fourth larval instar was extracted with the PureLink Genomic DNA Mini Kit (Invitrogen, Grand Island, New York, US) according to the manufacturer’s instructions. A 450-bp fragment of the ND5 gene of 86 individuals from seven populations (Table 1) was amplified by polymerase chain reaction (PCR), using N5A and N5B primers [12]. The amplified products were analyzed by electrophoresis in 1% (w/v) agarose gels in tris-acetate buffer and ethidium bromide staining, and the purified PCR products were submitted for nucleotide sequencing (Macrogen, Korea). The identity of the DNA sequence datasets was confirmed by nucleotide Basic Local Alignment Search Tool (BLASTn). Sequences were manually inspected and corrected using the program Chromas Lite version 2.2.1 (Technelysium, South Brisbane, Australia) and aligned using Multiple Sequence Comparison by Log-Expectation (MUSCLE) [17]. Haplotype frequencies for each population were calculated using the program DNAsp 5.10 [18]. The results obtained were compared with the 14 haplotypes previously obtained to determine the distribution of mitochondrial lineages of *A*. *aegypti* populations present along Route N° 2 [12].

The dominant haplotype of this region (H1) was detected in all sampled areas, while haplotype H2 was present only in Chascomús and Lezama. According to Albrieu Llinás and Gardenal [12], H1 was a unique haplotype found in eight populations from the east of Argentina, and the presence of this haplotype would indicate a recent recolonization event of this mosquito after the major control campaign in this area. Moreover, the presence of this haplotype in the northwestern and northeastern regions indicates that the expansion areas of *A*. *aegypti* would be influenced mainly by nearby populations. On the other hand, haplotype H2 was found by Albrieu Llinás and Gardenal [12] in the northeast and center of Argentina. In our present study, H2 was detected only in the north of this new distribution, in Chascomús and Lezama, although at a low frequency (7% and 36%, respectively, Fig 1). This result suggests that H2 could have been introduced in these localities by a lower number of travelers from northern populations than those having H1 or that it has expanded not far from a recently colonized locality.

It has been discussed that the dispersal and colonization of new areas by *A*. *aegypti* and the viruses they transmit may be influenced by climatic conditions like global warming or a specific phenomenon, such as El Niño [19,20]. While the global phenomenon could be influencing the movement of these mosquitoes because it generates ideal conditions for their development (like mild temperature and water availability), there are factors on a smaller scale that could help the movement of *A*. *aegypti* on a local scale, like human transportation and the offer of different breeding containers, such as used tires and car batteries exposed to rainfall [21]. In fact, it is widely accepted that *A*. *aegypti* was introduced into America from Asia via slave ships [22]. In a previous study, we hypothesized that the dispersal of *A*. *aegypti* could be due to human activities increasing the mosquito dispersion in a passive way in the southeast of Argentina [14]. We detected established populations of *A*. *aegypti* in Lezama (at 39.2 km to Chascomús) in 2011 and then in Lezama, Castelli (27.7 km south from Lezama), and Dolores (31.8 km south from Castelli) in 2012; therefore, these mosquito populations commuted 59.5 km in only one year, which would imply a great influence of traffic flow to disperse populations at such a rapid rate.

In the present study, passive dispersal hypothesis is supported by the low diversity (*n* = 2) of haplotypes found in the studied range. As *A*. *aegypti* has a short flight range (about 10–800 m) during its entire lifetime [23], a high diversity of haplotypes between sampling points studied would be expected. However, we might think that in this expansion area there is a passive dispersal, probably due to the human movement between these locations, understanding human movement as not only commuting of people but also trading. Passive transport of eggs, larvae, and adults has been suggested as the main mechanism for long-distance dispersal not only by the terrestrial trade of used tires [9,24], other goods [25], and tourism [26] but also by other types of transportation like planes [27] and boats [28,29].

A recent colonization of some species present in new geographical regions because of anthropic action often results in low levels of genetic diversity [12,30]. The higher rate of travelers along all the roads increases the risk of mosquito transference and thus the probability of genetic exchanges between the insect populations. According to our results, only passive migration by human activity may explain the observed patterns, and it would be a very important factor, at local scales, for the colonization of new areas. Results presented here show, once again, the urgent need to implement effective campaigns to control vector mosquitoes—and consequently, a need for the development of responsible control campaigns for mosquito-borne diseases.
